# *PHD-finger* family genes in wheat (*Triticum aestivum* L.): Evolutionary conservatism, functional diversification, and active expression in abiotic stress

**DOI:** 10.3389/fpls.2022.1016831

**Published:** 2022-12-12

**Authors:** Fei Pang, Junqi Niu, Manoj Kumar Solanki, Shaista Nosheen, Zhaoliang Liu, Zhen Wang

**Affiliations:** ^1^ College of Agriculture, Yulin Normal University, Yulin, China; ^2^ Plant Cytogenetics and Molecular Biology Group, Faculty of Natural Sciences, Institute of Biology, Biotechnology and Environmental Protection, University of Silesia in Katowice, Katowice, Poland; ^3^ School of Agricultural Engineering and Food Science, Shandong University of Technology, Zibo, China

**Keywords:** *PHD-finger* genes, wheat, phylogenetic analysis, expression patterns, abiotic stress

## Abstract

Plant homeodomain (PHD) transcription factors (TFs) are a class of proteins with conserved Cys4-His-Cys3 domains that play important roles in plant growth and development and in response to abiotic stresses. Although characterization of *PHDs* has been performed in plants, little is known about their function in wheat (*Triticum aestivum* L.), especially under stress conditions. In the present study, 244 TaPHDs were identified in wheat using comparative genomics. We renamed them *TaPHD1-244* based on their chromosomal distribution, and almost all PHD proteins were predicted to be located in the nucleus. According to the unrooted neighbor-joining phylogenetic tree, gene structure, and motif analyses, *PHD* genes were divided into four clades. A total of 149 *TaPHD* genes were assigned to arise from duplication events. Furthermore, 230 gene pairs came from wheat itself, and 119, 186, 168, 7, 2, and 6 gene pairs came from six other species (*Hordeum vulgareto, Zea mays*, *Oryza sativa*, *Arabidopsis thaliana*, *Brassica rapa*, and *Gossypium raimondii*, respectively). A total of 548 interacting protein branches were identified to be involved in the protein interaction network. Tissue-specific expression pattern analysis showed that *TaPHDs* were highly expressed in the stigma and ovary during flowering, suggesting that the *TaPHD* gene plays an active role in the reproductive growth of wheat. In addition, the qRT-PCR results further confirmed that these *TaPHD* genes are involved in the abiotic stress response of wheat. In conclusion, our study provides a theoretical basis for deciphering the molecular functions of *TaPHDs*, particularly in response to abiotic stress.

## Introduction

Plants encounter various unfavorable growth conditions during their life cycle, such as pests and diseases, drought, and extreme temperatures. In response to adverse external environments, plants activate *in vivo* defense response mechanisms by inducing stress-responsive gene expression (Fujita et al., 2006; Zhu et al., 2019). Many plant-specific transcription factor (TF) family members are involved in plant-specific developmental processes and participate in and regulate the stress response of plants to the external environment, thereby improving their adaptation to adversity (Yamasaki et al., 2013). To date, some such transcription factors have been successively isolated from many species of plants, such as AP2/ERF (Mizoi et al., 2012), bHLH (Sun et al., 2018), MYB (Li et al., 2015), and WRKY (Rushton et al., 2010). Among these, the plant homeodomain (PHD)-finger transcription factor family is tissue-specific and plays an important role in plant growth, development, and transcriptional regulation by adversity. The PHD is a conserved zinc finger structural domain in biological evolution and is commonly distributed in eukaryotes ranging from yeast to plants and animals (Ogryzko et al., 1996; Gibbons et al., 1997; Kehle et al., 1998; Papoulas et al., 1998; Martin et al., 2006). A typical PHD domain consists of 50-80 amino acid residues with a characteristic Cys4-His-Cys3 sequence, which is arranged in a manner similar to RING (Cys3-His-Cys4) and LIM (Cys2-His-Cys5) (Aasland et al., 1995; Borden and Freemont, 1996). The most important function of the PHD domain is the specific recognition of various histone modifications and DNA sequences, thus acting in transcriptional regulation and participating in various biological processes in organisms (Li et al., 2006; Hu et al., 2011; Xi et al., 2011). For example, previous studies have shown that, in model plants, proteins containing PHD domains are involved in embryonic meristem germination, root development, photoperiod, vernalization, meiosis, and post-meiotic pollen development. PHD domains play an important role in plant growth and development (Mouriz et al., 2015).

PHD domains are a class of relatively small protein domains. Their relatively conserved cysteine and histidine can stabilize the normal spatial structure by binding zinc ions, so that the three-dimensional conformation of the entire domain is basically spherical (Kwan et al., 2003). In addition to the conserved Cys4-His-Cys3 residues, PHD proteins usually contain highly diverse sequences. These diverse sequences form genes with different biological functions within the *PHD-finger* family. For example, the PHD domain–containing protein MMD1 is involved in essential chromatin remodeling and transcriptional events during male meiosis (Yang et al., 2003). In *Arabidopsis*, the ALFIN1-like (AL) protein, which contains the PHD domain, plays a key role in seed germination (Molitor et al., 2014). Furthermore, the PHD-finger protein VIL1 is involved in the photoperiod and vernalization pathways, as it regulates the expression of related floral repressors (Sung and Amasino, 2004). ATX1 and ATX2 have histone methyltransferase activities and regulate the development of roots, leaves, and floral organs, as well as the transcription of some stress genes (Saleh et al., 2008).

Since Schinder first discovered and identified PHD proteins in plants (Schindler et al., 1993), an increasing number of *PHDs* have been reported. To date, 59 *Oryza stiva* members (Sun et al., 2017), 108 *Gossypium hirsutum* members (Wu et al., 2021), 72 *Solanum tuberosum* members (Qin et al., 2019), 60 *Phyllostachys edulis* members (Gao et al., 2018), and 67 *Zea mays* members (Wang et al., 2015a) have been identified. It is known that PHD proteins not only participate in the regulation of plant growth and development but also play an important role in stress response, especially to abiotic stresses such as salt, high-temperature, low-temperature, and drought stress. In rice, overexpression of the *OsPHD1* gene can significantly improve resistance to low-temperature, high-salt, and drought stress (Liu et al., 2011). Overexpression of the PHD-finger transcription factor gene *OsMsr16* can enhance salt resistance in rice plants (Zhang et al., 2016). Wei et al. also found that *Arabidopsis thaliana* transgenic plants overexpressing soybean *GmPHD2* exhibited higher salt resistance, possibly because overexpression of *GmPHD2* enhanced the scavenging of oxidative substances (Wei et al., 2009). Furthermore, under abiotic stress, genes in the *PHD-finger* family in maize, cotton, and poplar show differential expression under salt, drought, and cold stress (Wang et al., 2015a; Wu et al., 2016; Wu et al., 2021). Thus, it can be seen that the *PHD* family genes play a crucial role in regulating plant resistance to stress.

Wheat is a major food crop worldwide and plays a crucial role in global food security. It is especially important to tap important resistance genes, breed new resistant wheat varieties, and improve the resistance of wheat itself (He et al., 2011). The *PHD-finger* gene family, which is essential for growth and development, has been identified and studied in many crops, but no systematic studies of the *PHD* gene family in wheat have been performed. In the present study, we identified *PHD-finger* family members in wheat for the first time and performed a comprehensive and systematic genome-wide analysis, including gene conserved motif analysis, phylogenetic relationships, Gene Ontology (GO) annotation analysis, covariance analysis, reciprocal relationship analysis, and subcellular localization. We also investigated the expression of PHD family proteins during growth and development, their specific expression in each organ, and their expression under multiple stresses of low temperature, high temperature, and drought. We lay the foundation for analyzing the functions of PHD proteins and regulating stress resistance and also provide theoretical references for the excavation of stress resistance genes and stress resistance breeding in wheat.

## Materials and methods

### Identification and classification analysis of *PHD* family genes in wheat

To identify PHD gene family members from wheat, whole genome data for *T. aestivum* (IWGSC RefSeq_v1.1) were obtained from the Ensembl plant database (http://plants.ensembl.org/info/website/ftp/index.html), and the PHD-finger domain (PF00628) was downloaded from the PFAM database (https://pfam.xfam.org/). The PHD protein sequences from *A. thaliana* (70) and *O. sativa* (59) (**Supplementary Table S1**) (Sun et al., 2017) were used as query sequences to search against the wheat protein dataset using the BLASTP program, and the threshold was set as E-value *<* 1e-5. The NCBI-Batch CD-Search (Marchler-Bauer et al., 2017) (https://www.ncbi.nlm.nih.gov/Structure/bwrpsb/bwrpsb.cgi), PFAM database, and SMART database (http://smart.embl.de/) were used to further confirm the candidate *PHD-finger* genes of *T. aestivum.* There were other spliced transcripts in the candidate genes of these species, and we selected the first splice variant as a representative for subsequent analysis.

The protein sequences of TaPHDs were computed using the ExPASy server (Artimo et al., 2012) to obtain the theoretical isoelectric point (pI), molecular weight (MW), instability index (II), aliphatic index (AI), and grand average hydrophobicity (GRAVY). Plant-mPLoc (Chou and Shen, 2010) (http://www.csbio.sjtu.edu.cn/cgi-bin/PlantmPLoc.cgi) and BUSCA (Savojardo et al., 2018) (Bologna Unified Subcellular Component Annotator, http://busca.biocomp.unibo.it) were used to predict the subcellular localization of the TaPHD proteins.

### Phylogenetic analyses of *TaPHD* genes

The PHD-finger protein sequences of *T. aestivum*, *A. thaliana*, and *O. sativa* were used for phylogenetic analysis. Jalview 2.11 software (http://www.jalview.org/) with the MUSCLE method with default parameters was utilized to conduct multiple sequence alignment. Evolutionary analysis involved 342 amino acid sequences (all wheat *PHD* genes, and most rice and *Arabidopsis PHD* genes). These analyses were conducted in MEGA X (Kumar et al., 2018) using the neighbor-joining method (Saitou and Nei, 1987). The percentage of replicate trees in which the associated taxa clustered together in the bootstrap test (1000 replicates) is shown next to the branches. The evolutionary distances were computed using the Poisson correction method and were expressed as the number of amino acid substitutions per site. The iTOL website (http://itol.embl.de/) was used to visualize the phylogenetic tree.

### Gene duplication and Ka/Ks analysis of *TaPHD* genes

MCScanX software (Wang et al., 2012) was used to detect collinear regions between *TaPHD* genes as well as collinear blocks of *TaPHDs* with three monocotyledons (*H. vulgareto*, *Z. mays*, and *O. sativa*) and three dicotyledons (*A. thaliana*, *B. rapa*, and *G. raimondii*). Whole genome data for *H. vulgareto*, *Z. mays*, *O. sativa*, *A. thaliana*, *B. rapa*, and *G. raimondii* were obtained from the Ensembl plant database (http://plants.ensembl.org/info/website/ftp/index.html). All *TaPHD* genes were mapped to their respective loci in the wheat genome in a circular diagram using shinyCircos (Yu et al., 2018). Gene duplication events of *TaPHDs* and synteny relationships between the aforementioned species were visualized using TBtools (v1.082) (Chen et al., 2020). The Ka/Ks values (non-synonymous substitution rate/synonymous substitution rate) were calculated after identification of duplicated genes, using the method of Nei and Gojobori as implemented in KaKs_calculator (Zhang et al., 2006) based on the coding sequence alignments. Subsequently, the divergence time of collinear gene pairs was calculated using the duplication events formula T = Ks/(2λ × 10^-6^) in millions of years (Mya), with λ = 6.5 × 10^-9^ (Wang et al., 2015b).

### GO annotation and protein-protein interaction network analysis of *TaPHD* genes

GO annotation of TaPHD proteins was available from the KOBAS database (http://kobas.cbi.pku.edu.cn/kobas3) (Xie et al., 2011). The full-length amino acid sequences of TaPHD proteins were uploaded to the original program, followed by drawing and annotation. GO annotations were performed for three types of analyses: biological processes, molecular functions, and cellular composition. The GO annotation results were visualized using the online tool OmicStudio (https://www.omicstudio.cn/tool) (Ye et al., 2018). All the predicted TaPHD proteins were submitted to the STRING database (https://string-db.org/cgi/input.pl). The minimum required interaction score was set to a high confidence (0.700). The maximum number of interactors was no more than 10 on the first shell.

### Expression of *TaPHD* genes

Transcriptional data for *TaPHDs* were obtained from the wheat expression website (http://www.wheat-expression.com/download) (Borrill et al., 2016; Ramírez-González et al., 2018) and were used to explore the potential biological functions of *TaPHD* genes in growth and development, abiotic and biotic stress, and other conditions. Systematic clustering analysis was performed based on the log2 of transcripts per million (TPM) values for the 244 *TaPHD* genes. R was used to display the expression patterns in a heat map, and OmicStudio (https://www.omicstudio.cn/tool) was used to display the histogram, volcano plot, and Venn diagram.

### Quantitative real-time PCR analyses (qRT-PCR) of *TaPHD* genes in response to environmental stresses

In this study, the seeds of the hexaploid common wheat variety “Zhengmai 7698” were surface-sterilized with 2% hydrogen peroxide, rinsed thoroughly with distilled water, and germinated with water saturation at 25°C for two days in Petri dishes on three layers of filter paper. The young seedlings were transformed and grown in 1/2 Hoagland’s culture solution under a 14 h light (25°C)/10 h dark (20°C) photoperiod. When the wheat grew to two leaves and one heart, the plants were subsequently treated with 16% polyethylene glycol 6000. For cold stress, wheat seedlings were exposed to 4°C for 12 h. For heat stress, wheat seedlings were exposed to 40°C for 12 h. New leaves of the three seedlings were collected as biological replicates, and each treatment had three replicates.

Total RNA was extracted using RNAiso Reagent (TaKaRa, Beijing, China) and Cdna was synthesized using the RT Master Mix Perfect RealTime kit (TaKaRa, Beijing, China). Quantitative real-time PCR was performed using the CFX Touch™ Real-Time PCR Detection System (Bio-Rad Laboratories, Hercules, CA, USA) and the SG Fast Qpcr Master Mix (Sangon Biotech, Shanghai, China). Relative expression levels were determined using the 2^(-ΔΔCt)^ method (Livak and Schmittgen, 2001), and β-actin was used as the internal control to normalize the expression levels of *TaPHD* genes. Specific primers used for qRT-PCR are listed in **Supplementary Table S2**.

### Determination of subcellular localization of TaPHD11, TaPHD19, and TaPHD133

Full-length open reading frames of *TaPHD11, TaPHD19*, and *TaPHD133* were obtained from “Zhengmai 7698” Cdna (**Supplementary Table S2**). The Coding sequence (CDS)of *TaPHD11, TaPHD19*, and *TaPHD133* were cloned into the pJIT16318 vector at the BamHI site using specific primers (**Supplementary Table S2**). The pJIT16318 vector contained a CaMV 35S promoter and C-terminal GFP. Transient expression assays were conducted as described by Cui et al. (2019). Approximately 4 × 10^4^ mesophyll protoplasts were isolated from 12-day-old wheat seedlings. The transfected protoplasts were incubated at 23°C for 12 h. The GFP fluorescence in the transformed protoplasts was imaged using a confocal laser-scanning microscope (LSM 700; Zeiss).

## Results

### Identification and classification analysis of *PHD* genes in wheat

In this study, 244 *T. aestivum* genes were designated *PHD* genes with two query methods; HMM and BLASTP were used for identification, and three websites, NCBI-Batch CD-Search, PFAM database, and SMART database, were used for confirmation (**Supplementary Table S3**). These *PHD* genes were renamed *TaPHD1* to *TaPHD244*, based on the order of their chromosomal locations and physical positions.

To further determine the characteristics of *TaPHD* genes, the ExPASy Server online tool was used to analyze the protein characteristics (**Supplementary Table S3**). The shortest protein contained 216 amino acids (*TaPHD158, TaPHD175*) and the longest protein contained 2853 amino acids (*TaPHD204*); the molecular weight was between 24567.82 Da (*TaPHD158*) and 310347.53 Da (*TaPHD204*). The protein instability index showed that all *PHD* genes were unstable proteins. The isoelectric point of *TaPHD* genes varied markedly from 4.42 (*TaPHD36*) to 9.65 (*TaPHD78*), and the aliphatic index varied significantly from 48.13 (*TaPHD26/39/51*) to 97.51 (*TaPHD42*). The GRAVY of TaPHD proteins in wheat varied from 0.016 (*TaPHD160*) to -1.285 (*TaPHD23*), indicating that they were all hydrophilic proteins, except for *TaPHD160* (**Supplementary Table S3**). We used two methods (Plant-mPLoc and BUSCA) to predict the subcellular localization of the TaPHD proteins. The results showed that a few TaPHDs may be localized in the chloroplast, mitochondrion, or cytoplasm, and most members were predicted to be located in the nucleus (**Supplementary Table S3**).

### Multiple sequence alignment and phylogenetic analysis of *PHD* genes

**Figure 1:**
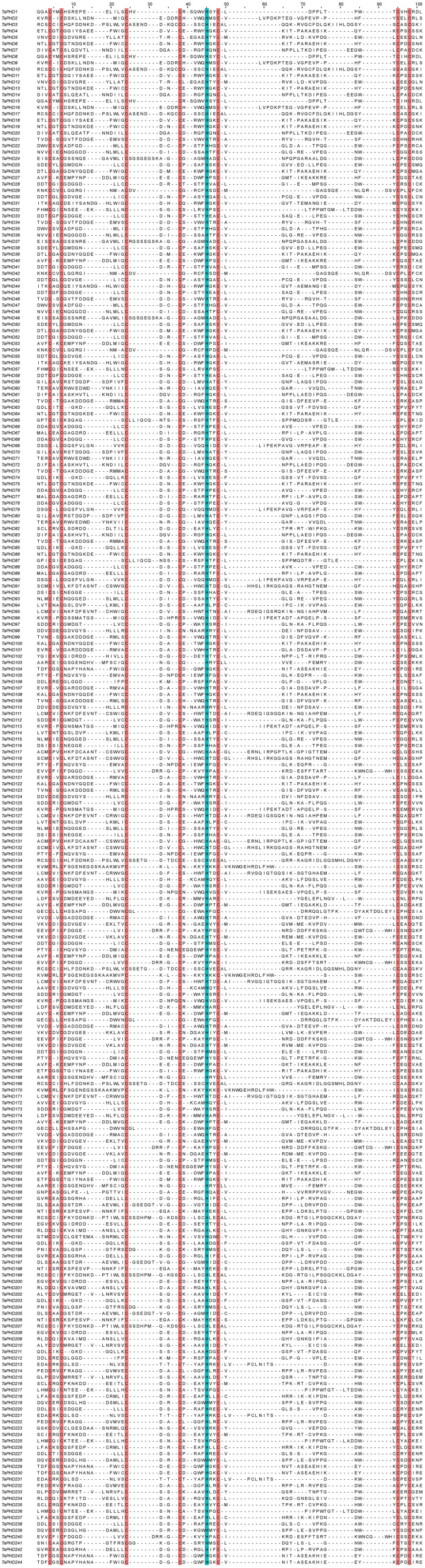
Protein sequence multiple alignment of the PHD-finger domains in TaPHD family proteins. The multiple alignment was conducted with the amino acid sequences within the predicted PHD domains by using Jalview software. The conserved amino acids (Cys4-His-Cys3) within the PHD-finger domains are shaded in red and blue.

Multiple sequence alignments of PHD domains were performed (**Figure 1**). Approximately 60 amino acids (aa) comprised a PHD domain containing basic Cys4-His-Cys3 sequence motifs in each TaPHD.

**Figure 2:**
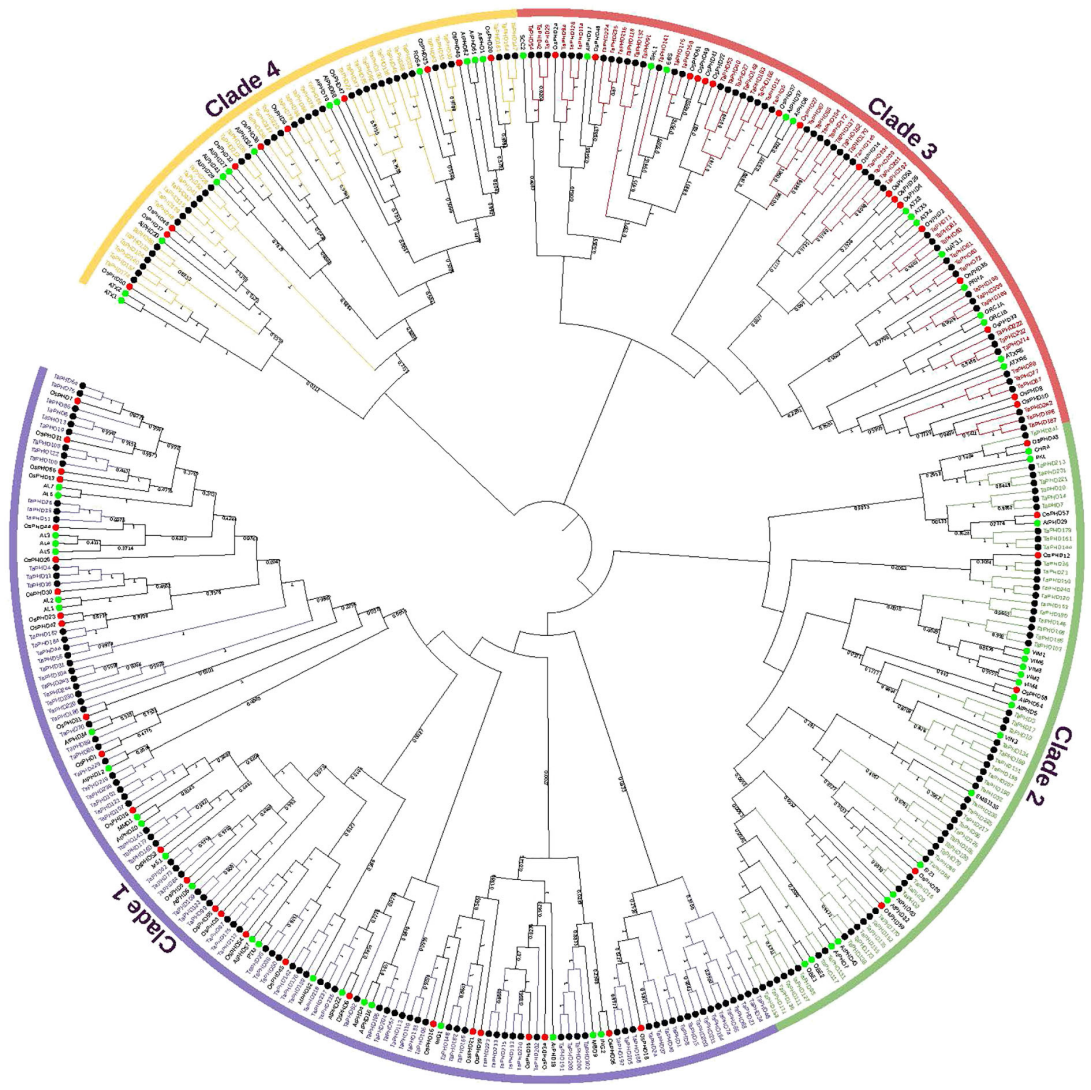
Phylogenetic tree of *PHD* genes in wheat, rice, and *Arabidopsis*. The tree was analyzed in MEGA X by using the neighbor-joining method. The PHDs from wheat, rice, and *Arabidopsis* are distinguished with black, red, and green dots. The PHD proteins were grouped into four distinct clades (clades 1-4), which are indicated by colored branches.

To evaluate the evolutionary relationships of *PHD* genes in *T. aestivum*, *O. sativa*, and *A. thaliana*, a neighbor-joining phylogenetic tree was constructed using full-length PHD proteins (**Figure 2** and **Supplementary Table S1**). Phylogenetic analysis showed that *PHD* family proteins can be divided into four clades (clades 1 to 4). *TaPHD* members were found in all clades. Clade 1 was the largest, with 95 *TaPHD* members, and clade 4 was the smallest, with only 38 members. The results showed that there were many small branches under each clade, and almost every small branch had corresponding genes of rice and *Arabidopsis*. This indicates that the *TaPHD* gene is not an evolutionary characteristic of monocotyledonous and dicotyledonous plants, and that the *PHD* gene family was formed before the differentiation of these two types of plants.

Protein domains are often functional carriers. According to phylogenetic and domain analyses (NCBI-Batch CD-Search, PFAM, and SMART database), 30 dominant types were identified in all wheat PHD proteins (**Table 1**). The results showed that among all wheat PHD proteins, 43 contained a typical PHD domain. The next most common, the jas-PHD and alifn-PHD domains, had 28 and 25 members, respectively; the PHD-Oberon_cc domain and the PHD-RING domains had 11 members, and the remaining domain types had less than ten members. The results showed that wheat PHD proteins contained a canonical PHD domain or double PHD domains. Owing to their different domains, differentiation in function was achieved.

**Table 1:** Types, names, and numbers of wheat *PHD-finger* genes.

Domain type	Wheat triad	Rice orthologs	Arabidopsis thaliana orthologs	Gene number	Chr	Genomes
PHD	TaPHD1/TaPHD8/TaPHD15			3	1	ABD
	TaPHD5/TaPHD12			2	1	AB
	TaPHD21/TaPHD34/TaPHD46			3	2	ABD
	TaPHD62/TaPHD73/TaPHD84	OsPHD5		3	3	ABD
	TaPHD82			1	3	D
	TaPHD94/TaPHD114/TaPHD128	OsPHD24		3	4	ABD
	TaPHD218/TaPHD226/TaPHD237	OsPHD24		3	7	ABD
	TaPHD98/TaPHD110/TaPHD124			3	4	ABD
	TaPHD99/TaPHD109/TaPHD123	OsPHD55	AtPHD6	3	4	ABD
	TaPHD101/TaPHD107/TaPHD121	OsPHD19	MS1,MMD1	3	4	ABD
	TaPHD143/TaPHD160/TaPHD177	OsPHD52	MS1,MMD1	3	5	ABD
	TaPHD103			1	4	A
	TaPHD137/TaPHD154/TaPHD172			3	5	ABD
	TaPHD146/TaPHD163/TaPHD180	OsPHD58	AtPHD54	3	5	ABD
	TaPHD168/TaPHD185			2	5	BD
	TaPHD186	OsPHD11		1	5	D
	TaPHD192/TaPHD201/TaPHD209	OsPHD14,OsPHD37	AtPHD8,AtPHD37	3	6	ABD
PHD-PHD	TaPHD135/TaPHD152/TaPHD170	OsPHD59	AtPHD32,AtPHD40	3	5	ABD
	TaPHD144/TaPHD161/TaPHD178		AtPHD29	3	5	ABD
	TaPHD216/TaPHD224/TaPHD235	OsPHD48		3	7	ABD
Alifn-PHD	TaPHD4/TaPHD11/TaPHD18	OsPHD30	AL1,AL2	3	1	ABD
	TaPHD6/TaPHD13/TaPHD19	OsPHD31	AL6,AL7	3	1	ABD
	TaPHD26/TaPHD39/TaPHD51	OsPHD44	AL3.AL4,AL5	3	2	ABD
	TaPHD31/TaPHD44/TaPHD56		AL1,AL2,AL3.AL4,AL5,AL6,AL7	3	2	ABD
	TaPHD64/TaPHD75/TaPHD86	OsPHD7	AL6,AL7	3	3	ABD
	TaPHD100/TaPHD108/TaPHD122	OsPHD56	AL6,AL7	3	4	ABD
	TaPHD167/TaPHD184	OsPHD23,OsPHD42	AL1,AL2	2	5	BD
	TaPHD104/TaPHD229/TaPHD230/TaPHD243/TaPHD244		AL1,AL2,AL3.AL4,AL5,AL6,AL7	5	4(7)	A(DD)UU
ARID-PHD	TaPHD142/TaPHD159/TaPHD176			3	5	ABD
RING-PHD	TaPHD23/TaPHD36/TaPHD48	OsPHD46	AtPHD30	3	2	ABD
	TaPHD93/TaPHD115/TaPHD129	OsPHD17	AtPHD30	3	4	ABD
ING-PHD	TaPHD105/TaPHD119/TaPHD133	OsPHD16	ING1	3	4	ABD
	TaPHD148/TaPHD165/TaPHD182	OsPHD21	ING2	3	5	ABD
BAH-PHD	TaPHD27/TaPHD40/TaPHD53	OsPHD41	SHL1	3	2	ABD
	TaPHD141/TaPHD158/TaPHD175	OsPHD49,OsPHD51	EBS	3	5	ABD
	TaPHD149/TaPHD166/TaPHD183	OsPHD22	SHL1	3	5	ABD
Jas-PHD	TaPHD28/TaPHD41/TaPHD52	OsPHD40	AtPHD1,AtPHD61,AtPHD62	3	2	ABD
	TaPHD147/TaPHD164/TaPHD181	OsPHD20	AtPHD1,AtPHD61,AtPHD62	3	5	ABD
	TaPHD30/TaPHD43/TaPHD55	OsPHD25		3	2	ABD
	TaPHD33/TaPHD45/TaPHD58		ROS4	3	2	ABD
	TaPHD22/TaPHD35/TaPHD47	OsPHD47	AtPHD68,AtPHD70	3	2	ABD
	TaPHD92/TaPHD116/TaPHD130	OsPHD47	AtPHD68,AtPHD70	3	4	ABD
	TaPHD66/TaPHD76/TaPHD88	OsPHD9		3	3	ABD
	TaPHD68/TaPHD78	OsPHD9		2	3	AB
	TaPHD106/TaPHD212	OsPHD32	AtPHD24,AtPHD26,AtPHD27,AtPHD41	2	4(7)	A(A)
	TaPHD220/TaPHD227/TaPHD238	OsPHD38	AtPHD24,AtPHD26,AtPHD27,AtPHD41	3	7	ABD
DDT-PHD	TaPHD25/TaPHD38/TaPHD50	OsPHD45	DDP1,DDP2	3	2	ABD
	TaPHD97/TaPHD112/TaPHD125	OsPHD54	DDP3	3	4	ABD
	TaPHD138/TaPHD155/TaPHD173	OsPHD54	DDP3	3	5	ABD
zf-HC5HC2H-PHD	TaPHD59/TaPHD70/TaPHD80	OsPHD1		3	3	ABD
PHD-Oberon_cc	TaPHD91/TaPHD118/TaPHD132			3	4	ABD
	TaPHD95/TaPHD111/TaPHD127		OBE1,OBE2	3	4	ABD
	TaPHD136/TaPHD153/TaPHD171		OBE1,OBE2	3	5	ABD
	TaPHD117/TaPHD131			2	4	BD
PHD-FN3	TaPHD3/TaPHD10/TaPHD17		VIN3	3	1	ABD
	TaPHD134/TaPHD151/TaPHD169		VIN3	3	5	ABD
	TaPHD190/TaPHD199/TaPHD207		VIN3	3	6	ABD
PHD-SANT	TaPHD63/TaPHD74/TaPHD85			3	3	ABD
	TaPHD194/TaPHD203/TaPHD211			3	6	ABD
PHD-WHIM1	TaPHD102/TaPHD191/TaPHD200/TaPHD208		MBD9	4	6(4)	(A)ABD
PHD-SET	TaPHD67/TaPHD77/TaPHD89	OsPHD8	ATXR5,ATXR6	3	3	ABD
	TaPHD187/TaPHD196/TaPHD242	OsPHD10	ATXR5,ATXR6	3	6	AB(U)
PWWP-PHD-SET	TaPHD60/TaPHD71/TaPHD81	OsPHD2,OsPHD4	ATX3,ATX4,ATX5	3	3	ABD
PWWP-FYRN-FYRC-PHD-SET	TaPHD140/TaPHD157/TaPHD174	OsPHD50	ATX1,ATX2	3	5	ABD
PHD-BAH	TaPHD234			1	7	D
PHD-BAH-AAA	TaPHD214/TaPHD222/TaPHD232	OsPHD33	ORC1A,ORC1B	3	7	ABD
PHD-homeodomain	TaPHD7/TaPHD14/TaPHD20		PRHA	3	1	ABD
	TaPHD61/TaPHD72/TaPHD83	OsPHD35	HAT3.1	3	3	ABD
	TaPHD189/TaPHD198/TaPHD206			3	6	ABD
PHD-PLN03142	TaPHD65/TaPHD87			2	3	AD
	TaPHD195/TaPHD204/TaPHD241			3	6	ABD
PHD-RING	TaPHD2/TaPHD9/TaPHD16	OsPHD29	SIZ1	3	1	ABD
	TaPHD69/TaPHD79/TaPHD90		SIZ1	3	3	ABD
	TaPHD96/TaPHD113/TaPHD126		SIZ1	3	4	ABD
	TaPHD139/TaPHD156		SIZ1	2	5	AB
PHD-JmjC-PLU1	TaPHD219/TaPHD228/TaPHD239			3	7	ABD
AAA_34-PHD-Helicase_C_4	TaPHD32/TaPHD57	OsPHD27	EMB1135	2	2	AD
	TaPHD217/TaPHD225/TaPHD236	OsPHD27	EMB1135	3	7	ABD
PHD-zf-HC5HC2H-zf-HC5HC2H	TaPHD193/TaPHD202/TaPHD210	OsPHD15,OsPHD34	AtPHD18	3	6	ABD
	TaPHD215/TaPHD223/TaPHD233			3	7	ABD
BRCT-BRCT-PHD	TaPHD24/TaPHD37/TaPHD49	OsPHD18		3	2	ABD
	TaPHD188/TaPHD197/TaPHD205	OsPHD18		3	6	ABD
PHD-SWIB-GYF-Plus3	TaPHD120/TaPHD150/TaPHD240			3	4(5)	(A)BU
PHD-SWIB-Plus3-GYF	TaPHD145/TaPHD162/TaPHD179			3	5	ABD
PHD-Chromo-Helicase_C-DUF	TaPHD213/TaPHD221/TaPHD231		PKL	3	7	ABD
PHD-Cohesin_HEAT-Nipped-B_C	TaPHD29/TaPHD42/TaPHD54		EMB2773	3	2	ABD

To better understand why *PHD-finger* genes are abundant in the wheat genome, we analyzed the homoeologous groups in detail (**Table 2**). A total of 35.8% of wheat genes were present in homoeologous groups of three, also termed triads (A:B:D = 1:1:1) (Consortium et al., 2018). In contrast, 84.8% of the *PHD-finger* genes identified were present in triads (**Table 2**). Also, the percentage of *PHD-finger* genes with homoeolog-specific duplications was lower for *PHD-finger* genes than for all wheat genes (1.6% vs 5.7%; **Table 2**). Loss of one homoeolog, on the other hand, was less pronounced in *PHD-finger* genes (6.6% vs 13.2%; **Table 2**). Only four *PHD-finger* genes were orphans/singletons. Thus, the high homoeolog retention rate could partly explain the high number of wheat *PHD-finger* genes.

**Table 2:** Groups of homoeologous *PHD-finger* genes in wheat.

Homoeologous group (A: B: D)	All wheat genes^1^	Wheat *PHD-finger* genes (all)		
		Number of groups	Number of genes	% of genes^2^
1: 1: 1	35.8%	69	207	84.8
**n: 1: 1/1: n: 1/1: 1: n^3^**	5.7%	1	4	1.6
1: 1: 0/1: 0: 1/0: 1: 1	13.2%	8	16	6.6
Other ratios^4^	8.0%	3	11	4.5
**Orphans/singletons**	37.1%	4	4	1.6
**Not categorized^5^**	-	-	2	0.8
	99.8%		244	100.0

^1^According to IWGSC (2018). ^2^Percentage calculated with 244 genes. ^3^For n > 1. ^4^E.g., n:1:n or 0:1:n, n > 1. ^5^See **Table 1** and **Table S3**.

### Chromosomal location, gene duplication, and synteny analysis of *TaPHD* genes

**Figure 3:**
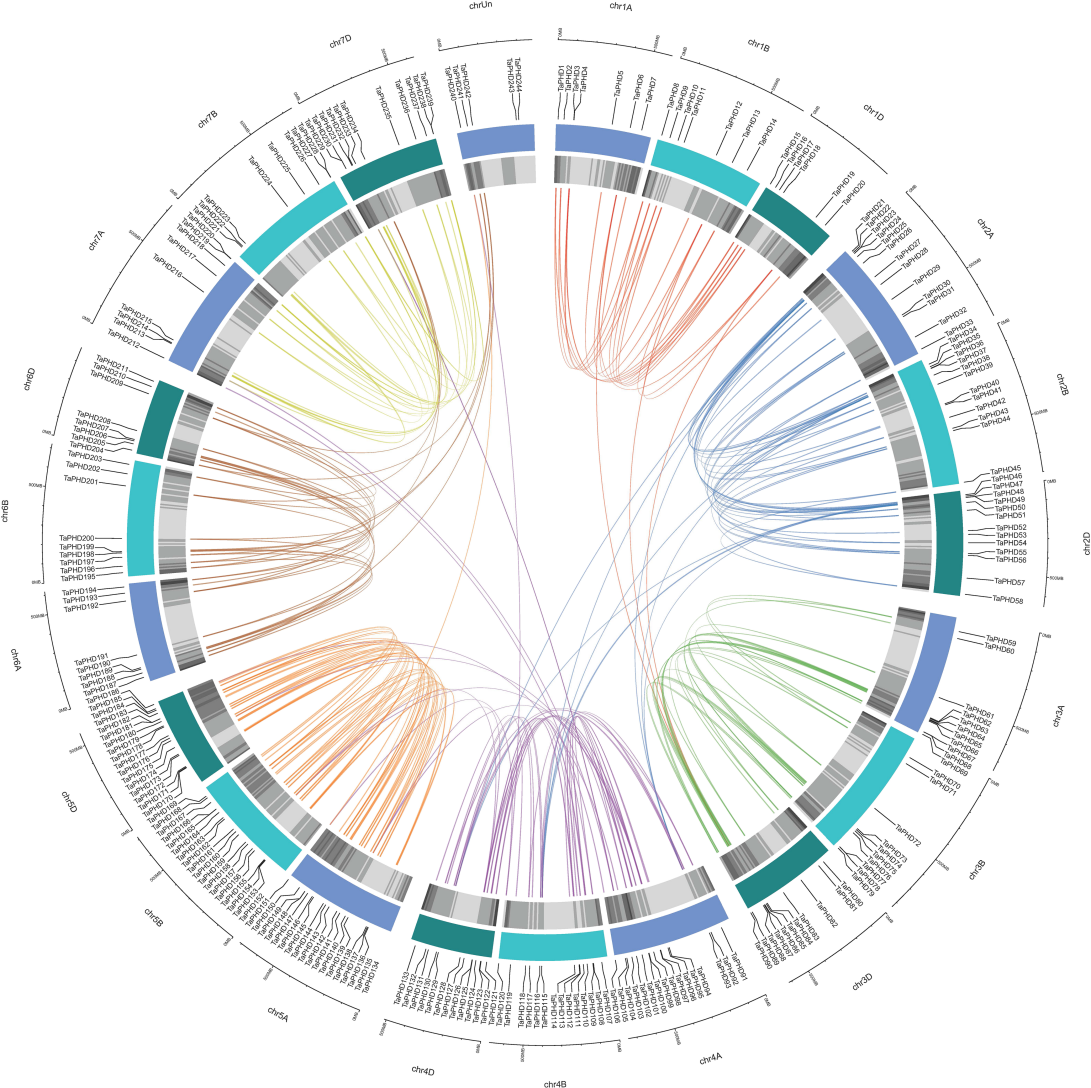
Chromosomal localization of *TaPHDs.* The light blue column represents the chromosome. The depth of blue in the columns represent the density of genes on the chromosome.

Based on the reference GFF3 files, the physical positions of *PHD* genes on the corresponding chromosomes are shown in **Figure 3**. The identified *TaPHDs* could be mapped on every chromosome and evenly across the three sub-genomes. The map shows that chromosomes 5B and 5D harbor the largest number of *TaPHD* genes (18), whereas chromosome 1D contains the least (6).

**Figure 4:**
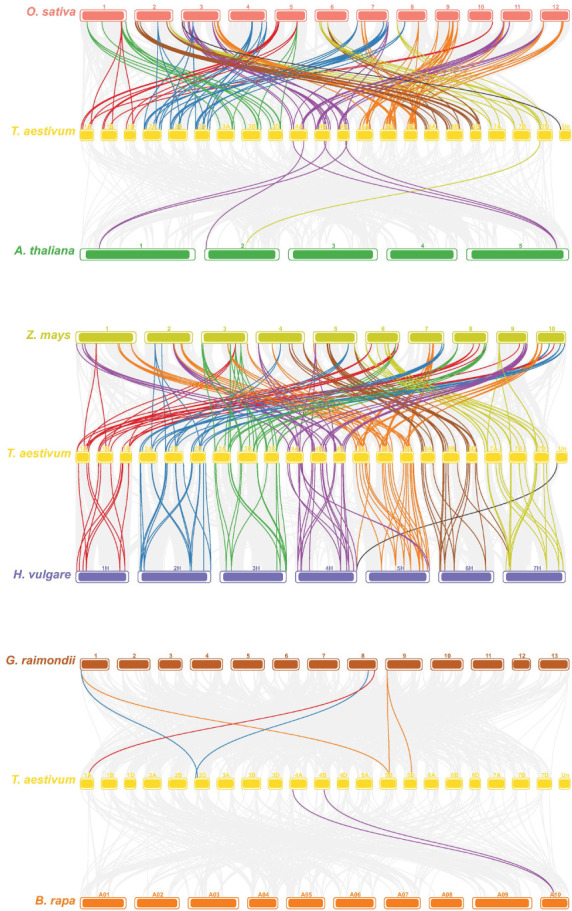
Synteny analysis of *PHD* genes in wheat. All *TaPHD* genes were mapped to their respective locus in the wheat genome in a circular diagram using shinyCircos (Yu et al., 2018). Subgenomes are indicated by different shades of blue (outer track), and chromosomal segments are indicated by shades of gray (inner track). Homoeologous *PHD* genes were inferred by phylogeny (for details see the Materials and Methods section) and linked with chromosome-specific colors.

**Figure 5:**
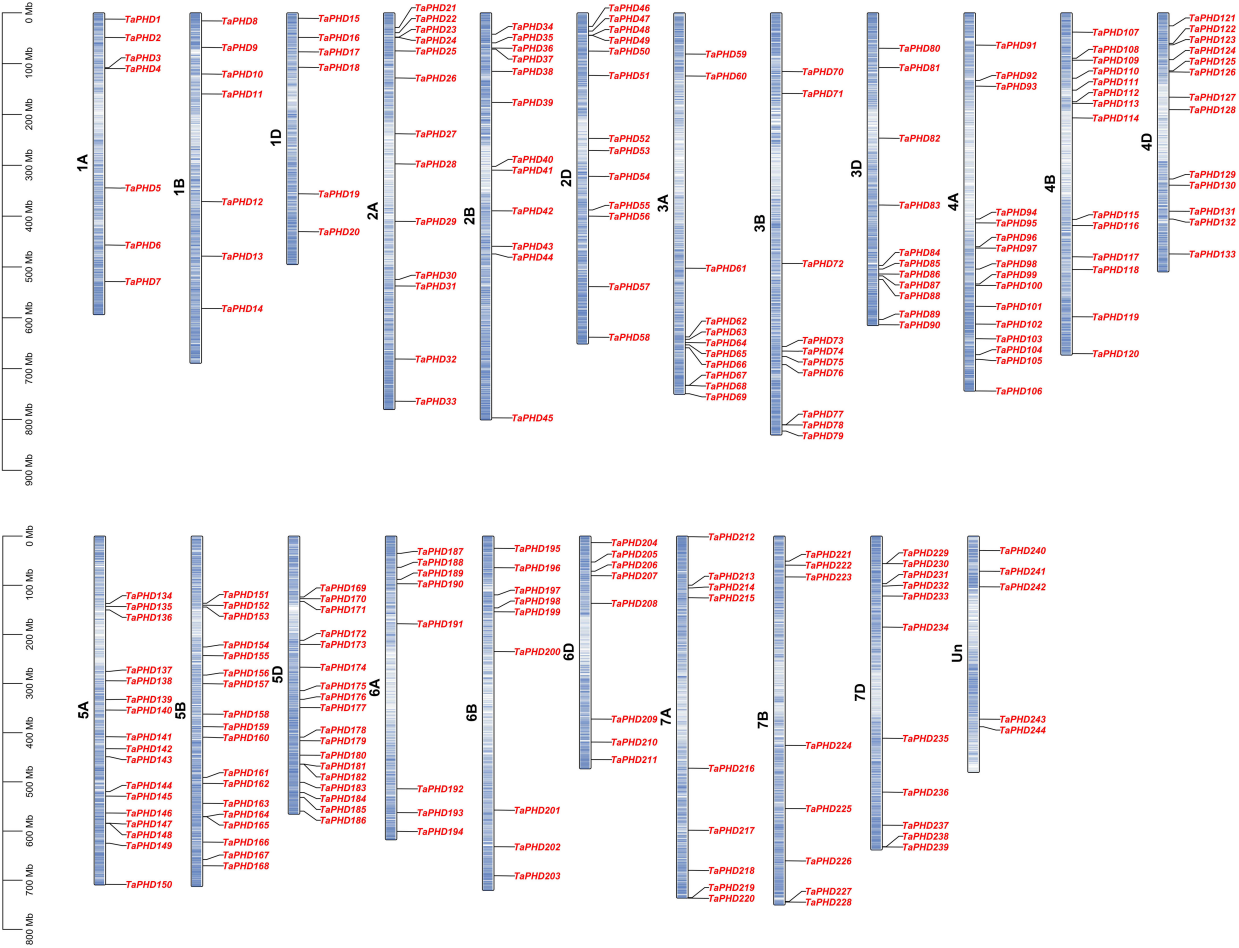
Synteny analysis of *PHD* genes between wheat and six representative plants (maize, barley, rice, *Arabidopsis*, cotton, and *Brassica rapa*). Each different species is replaced with a different color. The gray line in the background indicates a collinear block in the genome of wheat and other plants, while the line highlights the isomorphic *PHD* gene pair. Homoeologous *PHD* genes were inferred by phylogeny (for details see the Materials and Methods section) and linked with chromosome-specific colors.

Gene duplication is an indispensable mechanism by which organisms create new genes with similar or different functions (Song et al., 2019). Therefore, we analyzed the duplication events that occurred in the *TaPHD* gene family. A total of 230 *PHD* gene pairs from wheat were identified as duplicated (**Figure 4** and **Supplementary Table S4**). These similar *PHD* gene pairs had the same domain type and appeared in the same branch of the phylogenetic tree. Tandem and segment duplications are critical for the evolution of gene families to adapt to different environmental conditions. Interestingly, all the *TaPHD* gene pairs were associated with segmental duplication events. This suggests that this was the main route for expanding *PHD* genes in wheat and the many homologous genes on different wheat chromosomes suggest the high conservation of the family. To further infer the evolutionary origin and homology of the wheat *PHD* family, we constructed a collinear chart comparing six species with wheat, including three monocotyledons (*H. vulgareto*, *Z. mays*, and *O. sativa*) and three dicotyledons (*A. thaliana*, *B. rapa*, and *G. raimondii*) (**Figure 5** and **Supplementary Table S4**). We identified pairwise homologues of the *TaPHD* genes and detected 119, 186, 168, 7, 2, and 6 pairs of homologous genes from *H. vulgareto, Z. mays*, *O. sativa*, *A. thaliana*, *B. rapa*, and *G. raimondii*, respectively (**Figure 5** and **Supplementary Table S4**). This implies that *TaPHD* genes share a strong evolutionary relationship with *ZmPHDs*, *HvPHDs*, and *OsPHDs*. Furthermore, these results indicated that the *PHD* gene family was differentiated between monocotyledonous and dicotyledonous plants. This also indicated that *TaPHD* genes had a strong evolutionary relationship with *ZmPHDs*, *HvPHDs*, and *OsPHDs.* The average differentiation time was: barley (12.78 Mya) < rice (22.09 Mya) < maize (60.87 Mya).

Ka/Ks, the non-synonymous substitution ratio, determines the selection pressure for duplicated genes. According to the results (**Supplementary Table S4**), only a very few *TaPHD* gene pairs had Ka/Ks ratios >1, suggesting that the evolution of *TaPHD* genes was accompanied by strong purifying selection. The Ka/Ks ratios between wheat and three monocotyledonous plants were calculated based on the collinear gene pairs. Except for very few genes, the values of the other collinear gene pairs were all below 1, which confirmed that the evolution of the wheat *PHD* gene family underwent strong purifying selection. However, the Ka/Ks ratios of the collinear gene pairs between wheat and the three dicots could not be calculated properly. This is because most synonymous mutation sites have synonymous mutations; that is, the degree of sequence divergence and evolutionary distance is too large. Some *TaPHD* genes have formed at least five homologous gene pairs, such as *TaPHD9*, which may have played key roles in the evolution of the *PHD* gene family (**Figure 5** and **Supplementary Table S4**).

### GO annotation analysis and protein-protein interaction network of *TaPHD* gene*s*

**Figure 6:**
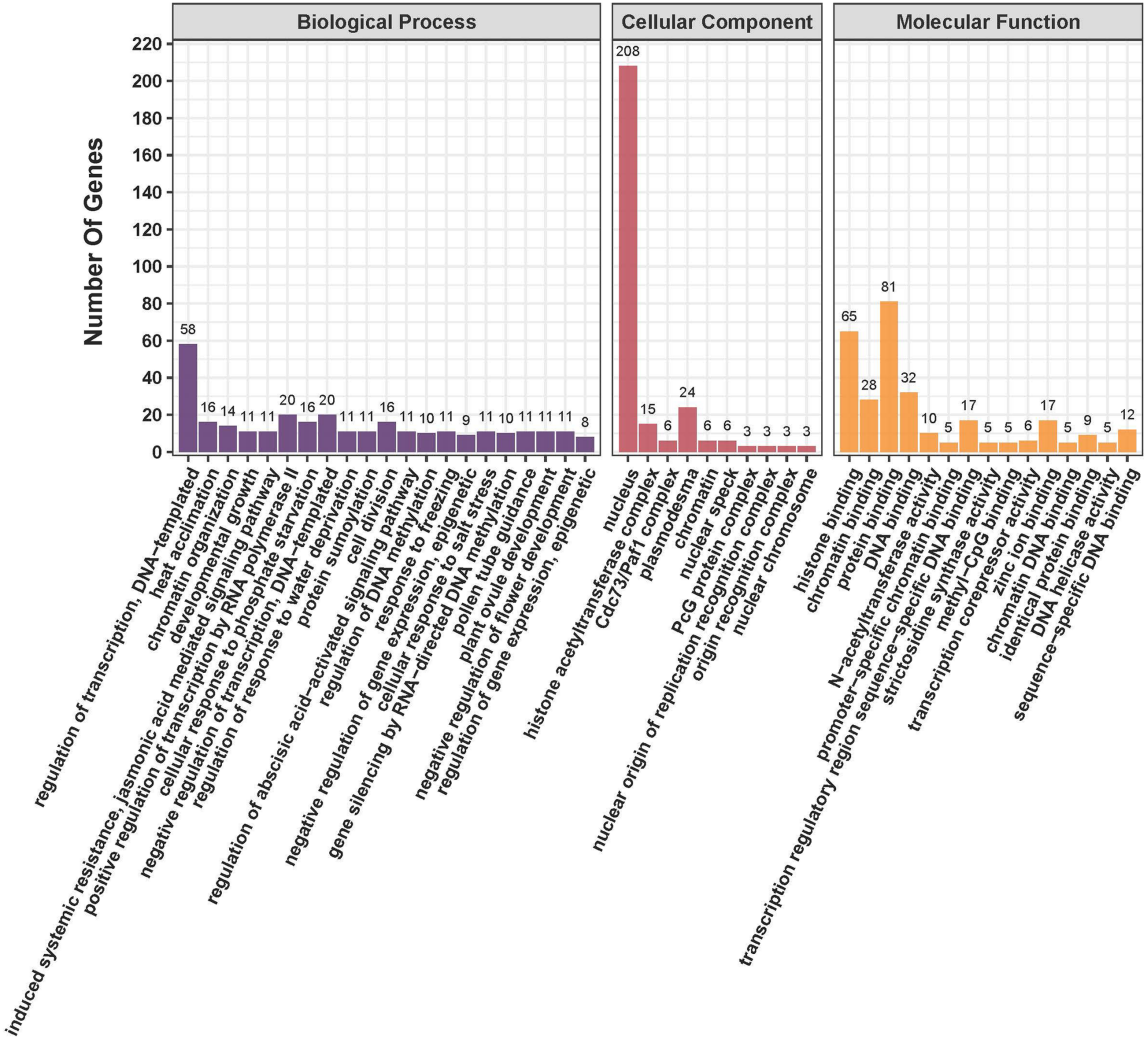
Functional annotation analysis of *TaPHD* genes. Gene Ontology (GO) classification based on *TaPHD* gene annotation. The GO terms are grouped into three main categories: purple for Biological Processes, red for Cellular Components, and yellow for Molecular Function.

We performed GO annotation analysis of the 244 TaPHD proteins, revealing that they may participate in a range of cellular components, molecular functions, and biological processes (**Figure 6** and **Supplementary Table S5**). The 244 TaPHD proteins were assigned a total of 105 GO terms. In biological processes, the three most highly enriched categories were related to the regulation of DNA-templated transcription, heat acclimation, and chromatin organization. Developmental growth and jasmonic acid-mediated systemic resistance were also particularly enriched. In the cellular component category, the most highly enriched categories were related to the nucleus, and 85% of the *TaPHDs* could participate in this process, whereas less than 10% of *TaPHDs* were involved in plasmodesma. Regarding molecular functions, the 65 most enriched *TaPHDs* were involved in histone binding, 28 *TaPHDs* were involved in chromatin binding, and 81 *TaPHDs* were related to protein binding.

**Figure 7:**
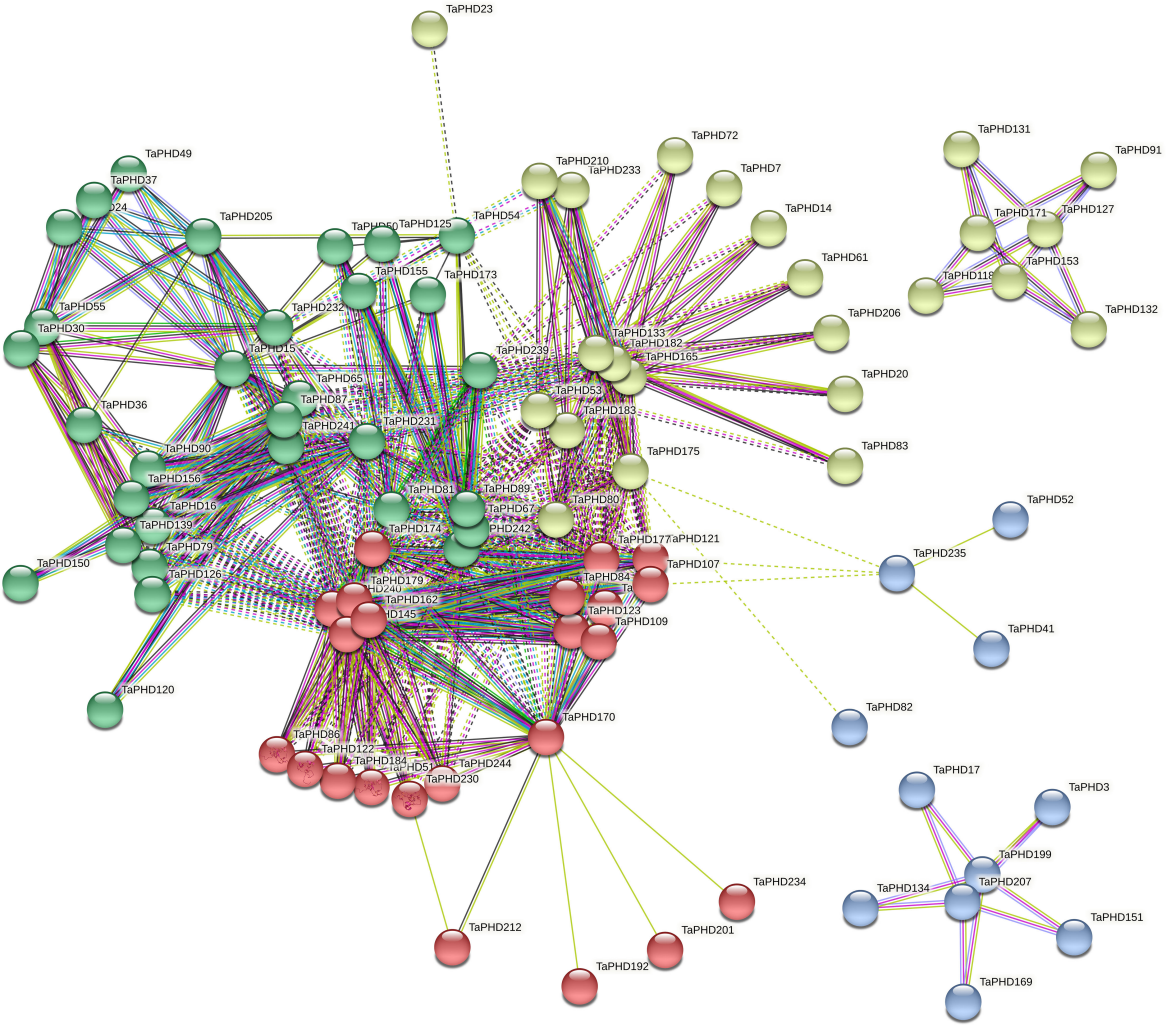
Predicted protein association networks analyses of TaPHD proteins. The four colors represent different interaction areas. The nodes represent the proteins, and the lines represent the protein-protein associations. Light blue and purple lines represent the known interactions from the curated database or experimentally determined interactions; green, red, and blue lines represent gene neighborhood, gene fusions, and gene co-occurrence, indicating that the proteins have the predicted interactions; yellow, black, and light blue lines represent textmining, co-expression, and protein homology, respectively.

To understand protein-protein interactions between TaPHDs and other proteins in wheat, we constructed a protein-protein interaction network (**Figure 7** and **Supplementary Table S6**). A total of 89 TaPHD proteins and 548 interacting protein branches were identified. According to the strength of the interaction, we divided the 89 proteins into four interaction regions, which are represented by different colors, as shown in **Figure 7**. Some TaPHDs, such as TaPHD15, TaPHD145, and TaPHD162, could interact with up to 28 proteins, suggesting that these TaPHD proteins play a significant role in the regulation of protein networks. Notably, we found that these proteins had a PHD domain or a PHD-SWIB-Plus3-GYF domain. Therefore, we believe that such domains are likely to play an important role in the PHD family.

### Expression analysis of *TaPHD* genes during growth and development

**Figure 8:**
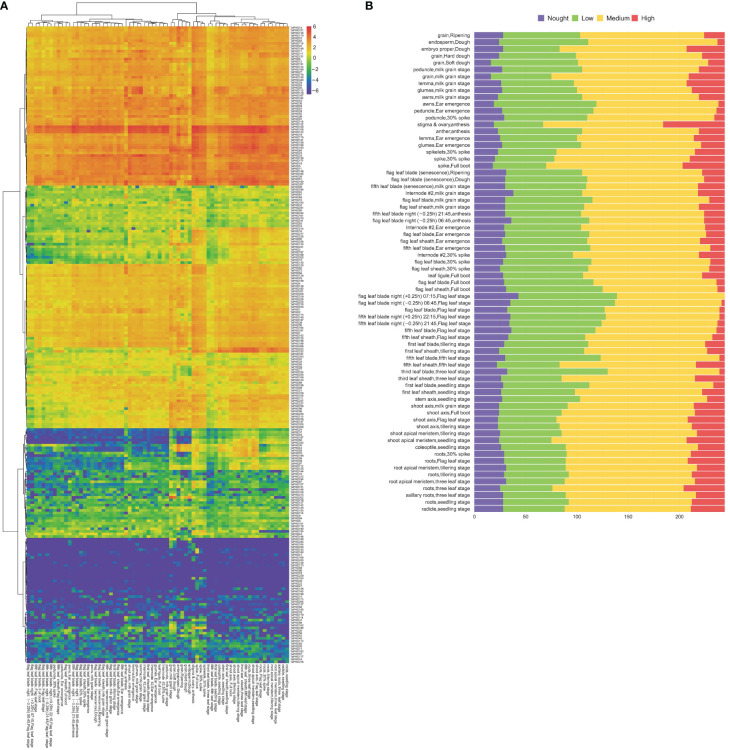
Transcriptome analyses of *TaPHDs* in different tissues. **(A)** Heat map of expression profiles for 244 *TaPHD* genes in different tissues. Red color indicates high expression levels; blue color indicates low expression levels. The gradual change of the color indicates different levels of gene log2-transformed expression. **(B)** Numbers of expressed genes in different tissues. High: TPM values >10, medium: 10 ≥ TPM values > 1, low: 1 ≥ TPM values > 0, none: TPM values = 0.

RNA-sequencing is a powerful tool for exploring certain gene transcription patterns using high-throughput sequencing methods (Wang et al., 2009). Systematic clustering analysis was performed based on the log2 of TPM values for 244 *TaPHD* genes (**Figure 8A** and **Supplementary Table S7**). The data showed that *TaPHD* gene expression showed great differences with the change in the growth period. In general, the expression of *TaPHDs* can be divided into three categories: the first group contains members that are widely expressed in many tissues under multiple developmental stage conditions; the second group contains those that are highly induced only at specific growth and development stages; and the last group includes members that do not appear to be expressed during growth and development. For example, *TaPHD100*, *TaPHD108*, and *TaPHD122* had high expression during most growth and developmental processes, except in the endosperm. There were also some genes (*TaPHD222* and *TaPHD232*) that had higher expression only in shoots and roots. Furthermore, some genes, such as *TaPHD68*, *TaPHD78*, and *TaPHD86*, were not expressed, which implies that these genes may have functional redundancy.

To further study the expression differences of this family in different stages and organs of wheat, we counted the number of high, medium, and low expression genes in each period and organ (**Figure 8B**). The data showed that the number of highly expressed genes was the largest in the stigma and ovary, reaching as high as 60, followed by a spike in the boot period, reaching 41. The lowest number of highly expressed genes (none) was found in the flag leaf blade at night in the flag leaf stage. Our results suggest that some *TaPHDs* may play important roles in many biological processes during wheat growth, especially during anthesis.

### Expression responses of *TaPHD* genes to abiotic/biotic stress

**Figure 9:**
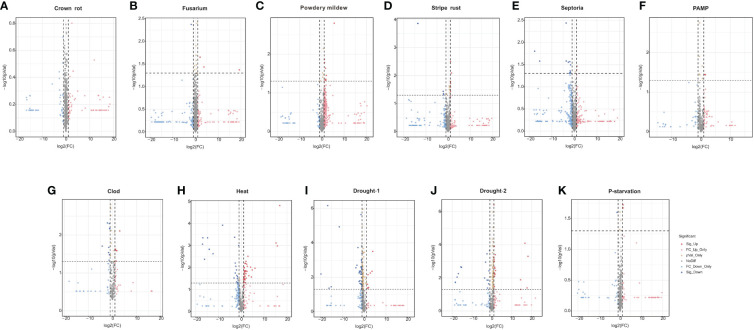
Expression of *TaPHDs* during different biological stress. Volcano map of expression profiles for 244 *TaPHD* genes under different biological/abiotic stresses, including **(A)** crown rot infection, **(B)** *Fusarium* infection, **(C)** powdery mildew infection, **(D)** stripe rust infection, **(E)** Septoria (*Septoria tritici* infection and *Zymoseptoria tritici* infection), **(F)** PAMP (chitin and flg22 infection), **(G)** cold stress, **(H)** drought-1 (drought stress in Giza 168), **(I)** drought-2 (drought stress in Gemmiza 10), **(J)** heat stress, and **(K)** P-starvation. DEGs were defined as Fold Change > 1 and FDR < 0.05.

The differential expression of *TaPHDs* under different conditions is shown in **Figures 9A–F** and **Supplementary Table S8**. During biological stress, we found that inoculation with *Fusarium*, powdery mildew, pathogen associated molecular patterns (PAMP), crown rot, *Septoria*, or stripe rust caused few changes in the expression of *TaPHD* genes. This suggests that *TaPHD* family members may not be associated with disease resistance.

**Figure 10:**
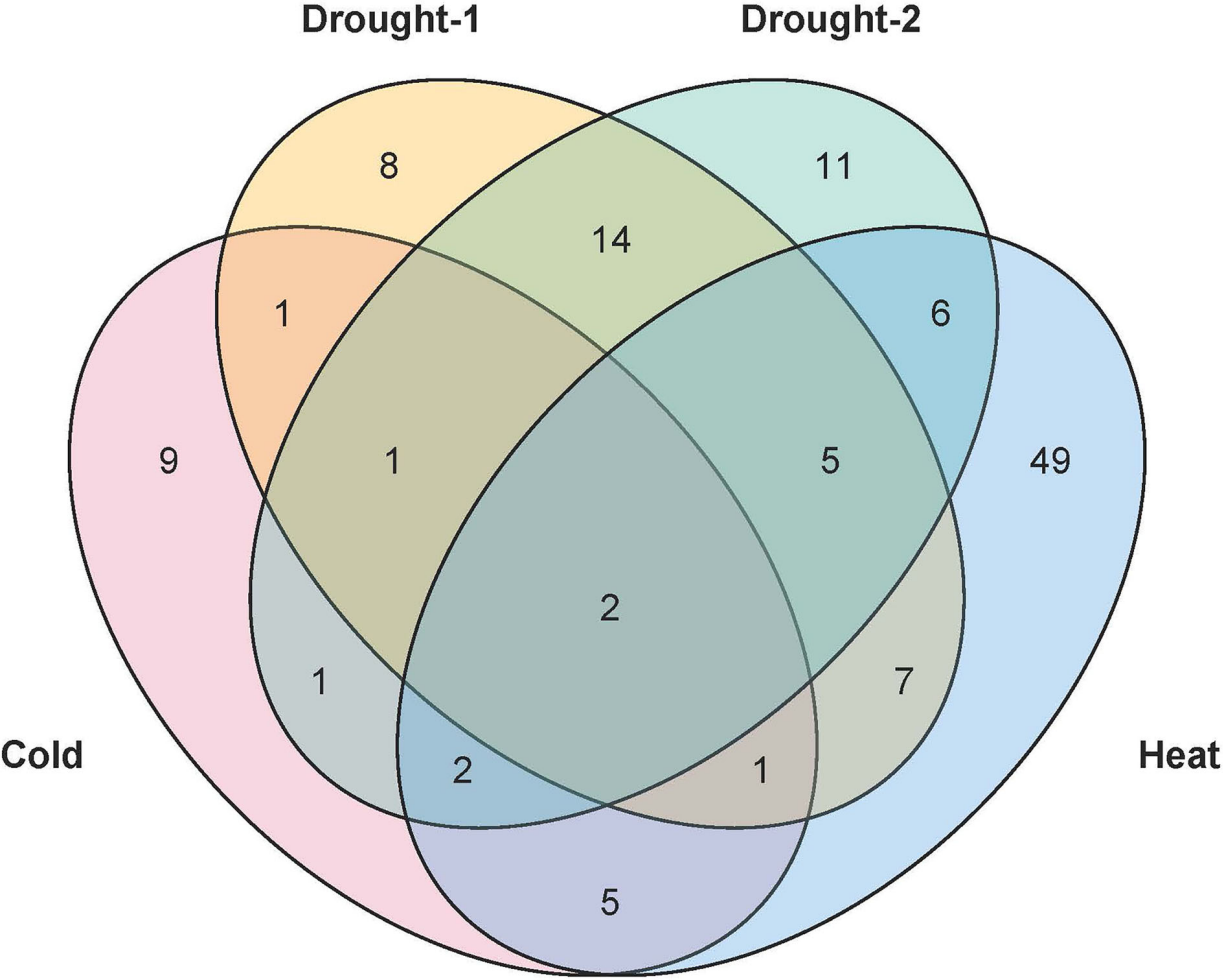
Venn diagram of DEGs in *TaPHD* genes during different abiotic stress. DEGs of *TaPHD* genes in different abiotic stress conditions, including cold stress, drought-1 (drought stress in Giza), drought-2 (drought stress in Gemmiza), and heat stress.

Under abiotic stress, there are many *TaPHD* genes whose expression changes are more obvious under high-temperature, drought, and cold conditions (**Figures 9G–K** and **Supplementary Table S8**). For example, after high-temperature treatment, the expression levels of many *TaPHD* genes (*TaPHD26*, *TaPHD75*, *TaPHD100, TaPHD115, TaPHD117*, and *TaPHD167*) were significantly altered compared to those in the experimental control group. In the drought starvation treatment, *TaPHD11, TaPHD19, TaPHD99*, *TaPHD141, TaPHD153*, and *TaPHD171* expression levels changed significantly. However, in the phosphorus starvation treatment, there were few changes in the expression of *TaPHD* genes. To further understand whether there is an intersection between the differential genes of the *PHD* family under drought, high-temperature, and low-temperature treatments, we drew a Venn diagram of DEGs in *TaPHD* genes during the four different transcriptomes (**Figure 10**, **Supplementary Table S9**). The data showed that *TaPHD215* and *TaPHD223* were significantly altered in every treatment. *TaPHD30, TaPHD96, TaPHD180, TaPHD174*, and *TaPHD239* gene expression varied greatly between the two drought and heat treatments. In addition, in cold and heat stress environments, the expression levels of five genes (*TaPHD109, TaPHD118, TaPHD120, TaPHD167*, and *TaPHD178*) were significantly changed.

### qRT-PCR confirmed the response capability of *TaPHD* genes to abiotic stress conditions

**Figure 11:**
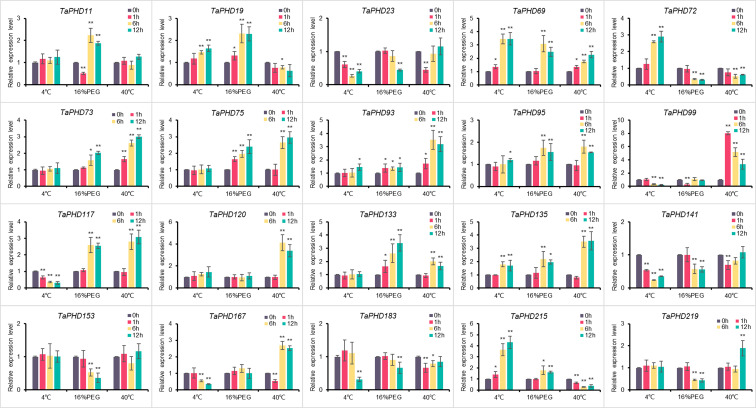
Relative expression levels of 20 genes under three different treatments. Expression of TaPHD genes in wheat were detected after 4°C, 16% PEG, and 40°C treatments for 0, 1, 6, and 12 h. Significant differences were determined by one-way ANOVA test: * p < 0.05; ** p < 0.01.

To elucidate the possible regulatory mechanisms of *TaPHD* genes under cold, drought, and heat conditions, we performed qRT-PCR analysis of 20 genes (**Figure 11**). The results showed that all 20 *TaPHDs* responded to different stress conditions and had different manifestations. Under low temperature stress induced by 4°C, the expression of five *TaPHD*s was significantly upregulated at different time points, and the expression of six *TaPHD*s was significantly downregulated at different time points compared with the control. In contrast, under 40°C-induced high-temperature stress, the expression of 12 *TaPHD*s was significantly upregulated at different time points compared with the control. The expression of five *TaPHDs* was inhibited at different time points. This indicated that compared with low temperature stress, high temperature stress could induce more changes in the expression of *TaPHD*s and could upregulate the expression more. In wheat under 16% PEG stress, the expression of ten *TaPHD*s was significantly upregulated at different time points. The expression of seven *TaPHD*s was inhibited at different time points. Among them, *TaPHD72* was most significantly inhibited, and it was downregulated four-fold at 6 and 12 h after treatment. The expression levels of *TaPHD69* and *TaPHD135* significantly increased after the three treatments. However, the expression levels of *TaPHD23* and *TaPHD141* significantly decreased after the three treatments. In addition, *TaPHD99* was strongly upregulated or downregulated by high temperature, low temperature, and PEG, and we speculated that this might be a key regulator of abiotic induction. In conclusion, we verified the effect of *PHD-finger* gene expression on the effect of three abiotic stresses in wheat using qRT-PCR. These results indicate that *PHD-finger* genes play an important role in coping with abiotic stress in wheat.

### Subcellular localization of TaPHD11, TaPHD19, and TaPHD133

**Figure 12:**
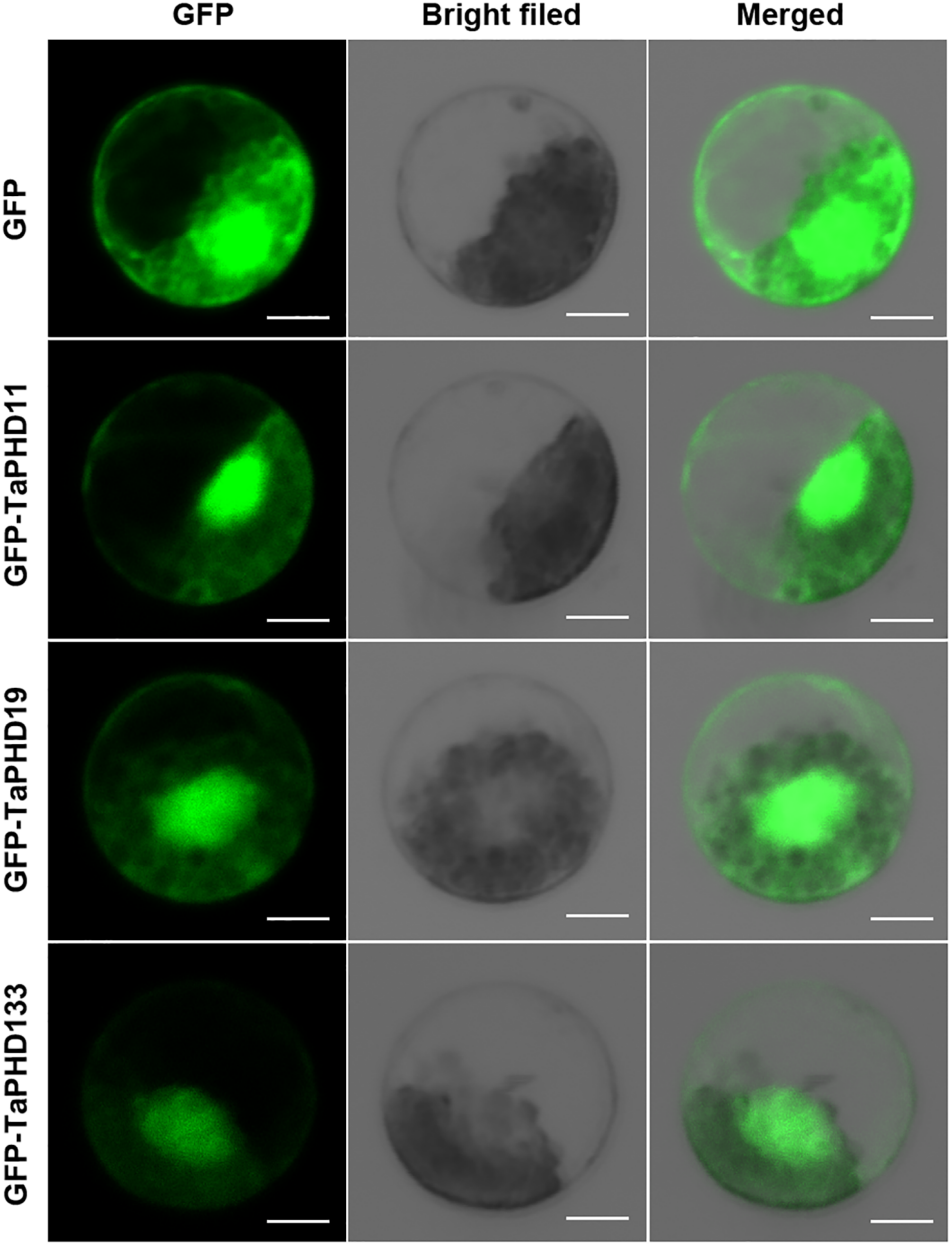
Subcellular location of *TaPHD11*, *TaPHD19*, and *TaPHD133*. Localization of TaPHD proteins under normal conditions. Images were observed under a confocal laser scanning microscope (LSM 700, Zeiss). Scale bars = 10 μm.

Previous studies have shown that most PHD finger proteins are localized in the nucleus, and only a few are localized in the membranes or other organelles (Gozani et al., 2003; Wu et al., 2016; Sun et al., 2017). For example, ZmPHD14 and ZmPHD19 are localized to the nucleus (Wang et al., 2015a). Also, GmPHD1 to GmPHD6 target the nucleus, and their nuclear localization requires the PHD domain (Wei et al., 2009). To better understand the functions of TaPHDs, we used Plant-mPLoc and BUSCA to predict their subcellular localization. The results showed that more than 90% of the TaPHD proteins were localized in the nucleus (**Table S1**). In *Arabidopsis thaliana*, the *PHD* genes *AL5* and *AL6* play a very important role in improving the resistance of plants to abiotic stress. Therefore, we selected TaPHD11 and TaPHD19, which are highly homologous to *AtALs*, for subcellular localization of wheat protoplasts. As shown in **Figure 12**, this suggests that, in wheat, the proteins TaPHD11 and TaPHD19 not only function in the nucleus but also in the membrane. In addition, research has shown that PHD finger ING2 is a phosphoinositide binding module and a nuclear PtdInsP receptor and suggests that PHD-phosphoinositide interactions directly regulate nuclear responses to DNA damage (Gozani et al., 2003). However, we studied the protein TaPHD133, which is highly homologous to ING1, and found that it is localized not only in the nucleus but also in the membrane. In summary, the subcellular localization of PHD proteins in wheat differs from that in other species.

## Discussion

As an important transcription factor in organisms, the *PHD* gene family not only plays a key role in regulating plant growth and development but also an important regulatory role when plants face biotic and abiotic stresses (Mouriz et al., 2015). In this study, we identified 244 *TaPHD* gene members in the wheat genome for the first time (**Supplementary Table S3**), which we divided into four large evolutionary branches. In terms of the number of genes, compared with the 59 and 67 *PHD* members in the diploid gramineous crops rice and maize, the *PHD* gene in wheat has a more exaggerated expansion and evolution. This is not only because the origin of wheat involves two polyploidy events, resulting in the existing allohexaploid bread wheat, but also because segmental duplication contributes to the amplification of *TaPHD* genes. Gene duplication events are important for the rapid expansion and evolution of plant gene families (Cannon et al., 2004). Approximately 70%–80% of angiosperms experience duplication events (Blanc et al., 2003; Bowers et al., 2003), and in common wheat (*Triticum aestivum* L.), more than 85% of the sequences are duplicates (Walkowiak et al., 2020). Our research revealed the presence of several segmental duplication events during the evolution of *TaPHD* genes (**Figures 4**, **5**). The proportion of *TaPHDs* with a 1:1:1 ratio of the three subgenomes A:B:D accounted for 84.8% of the total proportion (**Table 2**), which was much higher than the 35.8% observed for the whole wheat genome, indicating that the *PHD* gene family is highly conserved in the three subgenomes. When the *PHD* genes with different chaperone structural domains were subdivided (**Table 1**), the fold divergences were also different; for example, ING1, ING2, ROS1, EBS, and PKL were expanded 3-fold, while SHL1 was expanded 6-fold, and VIN3 and SIZ1 were expanded 9-fold and 11-fold, respectively. It is likely that the presence of many redundant genes has contributed to the stability of the genome of the hexaploid wheat species (Consortium et al., 2018). In terms of the covariance and evolutionary relationship of wheat *PHD* genes among species (**Figure 5**), the *PHD-finger* family diverged between monocotyledonous and dicotyledonous species, with the average divergence time from the monocotyledonous species in the order of barley (12.78 Mya) < rice (22.09 Mya) < maize (60.87 Mya), indicating a more similar genetic structure to barley in terms of *PHD* genes.

Genes perform their functions through transcription and translation, and the expression patterns of genes reflect their function. *PHD* genes can regulate the growth and development of plants. Therefore, their expression in different plant tissues has also attracted much attention. Studies have shown that the expression patterns of the *PHD* gene family in different species are concentrated in different tissue types (Sun et al., 2017; Qin et al., 2019; Wu et al., 2021). Most of them have a high level of expression in reproductive organs, including rice (Sun et al., 2017) and potato (Qin et al., 2019). However, in cotton, *GhPHD*s genes have the highest expression levels in ovule and fiber tissues (Wu et al., 2021). This study showed that the *TaPHD* expression in various tissues of wheat showed great differences with the growth period. In particular, *TaPHD* expression was highest in the stigma and ovary at the flowering stage. A large number of PHD proteins regulate plant reproductive and developmental processes, which indicates that *TaPHDs* may play the same role in rice and potato. It also has a similar expression pattern in Arabidopsis thaliana, the model plant with the most in-depth research. Some genes have been identified as having key functions. For example, MMD1, MS1, VIM1, and SHL1 in *Arabidopsis* have been shown to play key roles in the reproductive growth stage (Yang et al., 2003; Woo et al., 2007; Fernández Gómez and Wilson, 2014). Moreover, *TaPHD100*, *TaPHD108*, and *TaPHD122*, which were highly orthologous to *AtAL6* and *AtAL7* are highly expressed during the whole growth period. In *Arabidopsis*, *AtAL6* and *AtAL7* are methylated by histones *via* the PHD domain, and the modification sites H3K4me3 and H3K4me2 bind to regulate the expression of target genes. Alifn-PHD domain proteins bind to di- or trimethylated histone H3 (H3K4me3/2) and affect plant growth and development in *Arabidopsis* (Winicov, 2000). It can be seen that these three genes may play an important role in the growth and development of wheat *via* methylated histones. Furthermore, we can also speculate the function of the *PHD* gene in wheat through the expression mode of a more highly homologous *PHD* gene. PWWP-PHD-SET domain proteins have histone methyltransferase activities and regulate the development of roots, leaves, and floral organs, as well as the transcription of some stress genes (Saleh et al., 2008). Therefore, *TaPHD100*, *TaPHD108*, and *TaPHD122*, which have high coincidence with the PWWP-PHD-SET domain, may play important roles in regulating the growth and development of wheat histone methylation (Lee et al., 2009). In addition, *TaPHD222* and *TaPHD232* are only highly expressed in shoots and roots; these two genes are highly orthologous to ORC1A/B. In contrast, in *Arabidopsis*, the ORC1A/B protein binds methyl groups through the PHD domain and functions as a transcriptional activator (De La Paz Sanchez and Gutierrez, 2009). Therefore, we infer that *TaPHD222* and *TaPHD232* are essential for root and shoot development. However, their function during development requires further verification.

The PHD family not only regulates plant growth and development but also responds to abiotic stresses. Existing research shows that the *PHD* genes *AL5* and *AL6* in *Arabidopsis* bind to the promoter regions of downstream target genes, thereby inhibiting various signaling pathways to improve the resistance of plants to abiotic stresses such as low temperature, drought, and high salt (Gozani et al., 2003; Wei et al., 2015). Notably, in this study, *TaPHD11* and *TaPHD19*, which are highly homologous to *ALs*, were upregulated only under induction by PEG treatment. Through qRT-PCR analysis, we also found that *TaPHD11* and *TaPHD19*, which are highly homologous to *AtALs*, were significantly upregulated only under drought treatment. This finding is different from the results of the previous study in *Arabidopsis*, indicating that ALs seem to have different responses to abiotic stress in monocotyledonous and dicotyledonous plants. Meanwhile, subcellular localization experiments also showed that *TaPHD11* and *TaPHD19* were localized in the nucleus and cell membrane, indicating that they function not only in the nucleus but also in the cell membrane of wheat. This suggests that there are differences in the responses of *PHD* genes to abiotic stresses among species.

This does not mean that the PHD gene expression pattern of monocotyledons and dicotyledons is completely different*. TaPHD69*, which is highly homologous to *AtSIZ1*, can be significantly upregulated under low-temperature, drought, and high-temperature conditions. *AtSIZ1* accumulates high levels of SUMOylated proteins through an ABA-independent pathway in response to abiotic stresses such as drought, low temperature, and heat shock (Catala et al., 2007). The accumulation of *TaPHD69* seems to be beneficial for plants to cope with abiotic stress, which is similar to the function of *AtSIZ1* in *Arabidopsis*. In rice, the cis-acting elements DRE/CRT in the *OsPHD13* and *OsPHD52* promoters are upregulated by as much as 15-fold under low-temperature stress. Overexpression of *OsPHD1* can significantly improve plant resistance to stress (drought, high salt, and low temperature) (Liu et al., 2011; Ahmar and Gruszka, 2022). In maize, the expression of subfamily IX *TaPHD*s responds to salt, drought, and ABA stress (Wang et al., 2015a). Among *TaPHDs*, 45 *TaPHDs* genes were significantly changed under two or three treatments, indicating that *TaPHD*s play an active role in plant responses to low-temperature, drought, or high-temperature stress. *TaPHD117* was significantly upregulated under high-temperature and drought treatments and significantly downregulated under low-temperature treatment and had distinct expression patterns in response to different treatments. Therefore, whether *TaPHDs* act as key genes in the roots to cope with abiotic stress requires further verification, but our results suggest that *TaPHDs* have potential functions in plant responses to abiotic stress.

## Data availability statement

The datasets presented in this study can be found in online repositories. The names of the repository/repositories and accession number(s) can be found in the article/**Supplementary Material**.

## Author contributions

FP, ZL, and ZW designed the study. FP and MS conducted the experiments. JN and SN analyzed the data. FP, JN, ZL, and ZW wrote the manuscript. ZL and ZW revised and finalized the manuscript. All authors contributed to the article and approved the submitted version.
